# Novel eccentric corneoscleral donor preparation technique providing corneoscleral tectonic and central split corneal grafts for multiple recipients

**DOI:** 10.1007/s00417-021-05482-9

**Published:** 2021-10-30

**Authors:** Sigrid Roters, Alexander C. Rokohl, Ludwig M. Heindl, Claus Cursiefen

**Affiliations:** 1 Department of Ophthalmology, Faculty of Medicine and University Hospital of Cologne, University of Cologne, Kerpener Straße 62, 50937 Cologne, Germany; 2 Center for Molecular Medicine Cologne (CMMC), University of Cologne, Cologne, Germany


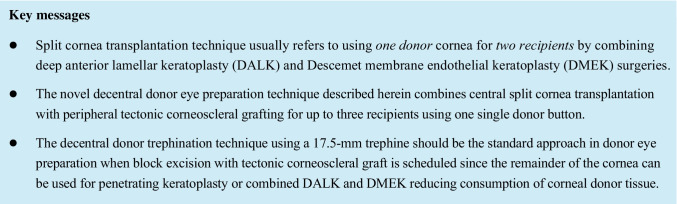


Dear Editor,

Today, split cornea technique is an established procedure and is mostly used for two recipients by combining deep anterior lamellar keratoplasty (DALK) and Descemet membrane endothelial keratoplasty (DMEK) surgeries [1–7]. However, for some surgical interventions including block excision with tectonic corneoscleral grafting, split cornea procedure is not planned regularly up to now [5, 8]. In the run-up for this procedure, normally a donor cornea with a bigger scleral ring is gained. Nonetheless, the preparation of the tectonic graft for covering the corneoscleral defect after block excision results in a rest donor cornea transplant which is normally too small for further regular size penetrating keratoplasties (PKs) or combined DALK/DMEK surgeries. However, using a modified donor transplant trephination technique, a corneoscleral transplant for regular size keratoplasties can be gained, also after preparation of a tectonic graft for block excision. Herein, we describe shortly this novel donor preparation technique, the differences compared to the standard procedure, possible applications, and the advantages and disadvantages for the first time.

**Fig. 1:**
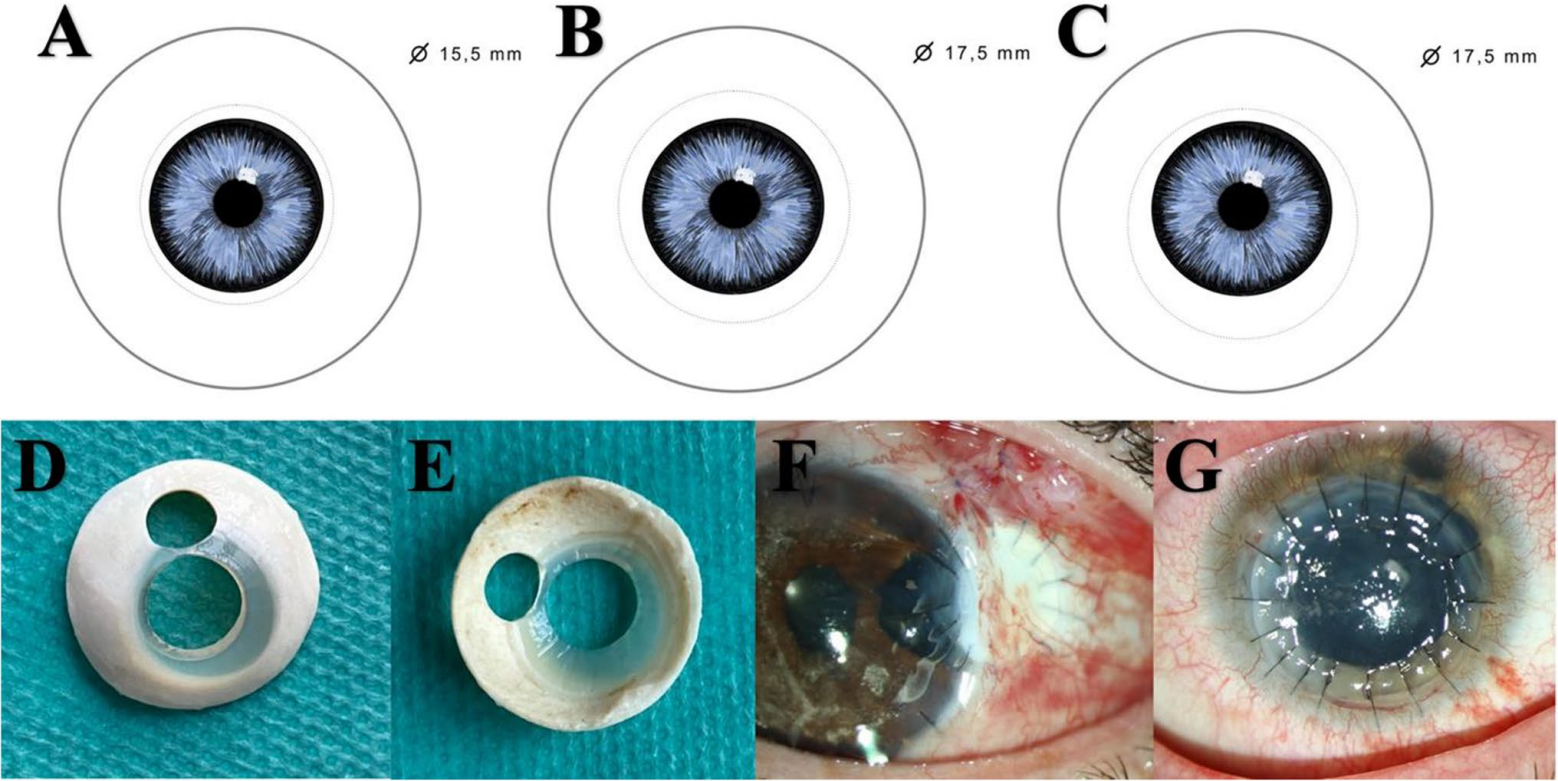
Decentral trephination of the donor corneoscleral button allows combined use for normal central DMEK and DALK in addition to a peripheral tectonic graft for corneoscleral grafting. Conventional central trephination of the donor eye using a trephine with 15.5 mm diameter (**A**) and a larger trephine of 17.5 mm (**B**). Decentral eccentric donor trephination also using a 17.5-mm trephine results in a bigger scleral area on one side of the donor transplant for up to 7.5-mm corneoscleral tectonic grafts (**C**). In addition to this peripheral corneoscleral tectonic graft, a full 7.5 or 8 mm or even larger diameter corneal graft can be gained for penetrating keratoplasty or combined DMEK/DALK (accidentally trephined minimal eccentric; **D**, **E**). Here, e.g., a 5.2-mm peripheral corneoscleral tectonic graft was used successfully for covering a 5 mm defect after block excision of a ciliary body tumor (first postoperative day, **F**), while a 7.5-mm corneal graft was used for an uncomplicated penetrating keratoplasty after perforated herpetic ulcer (second postoperative day, **G**)

Commonly for standard donor preparation (i.e., corneal grafts for DMEK, DALK, or PK except block excision), most cornea banks perform a central scleral trephination using a trephine with 15.5 mm diameter (Fig. 1A) [9]. For a block excision with a tectonic corneoscleral graft, a donor transplant with a bigger scleral ring is necessary. Therefore, usually, a central donor eye trephination is performed using a 17.5-mm trephine to gain a suitable corneoscleral grafting for this procedure (without regular size corneal splitting) (Fig. 1B). However, a *decentral* donor eye trephination also using a 17.5-mm trephine results in a corneoscleral donor transplant (Fig. 1C) which can be used both for block excision with tectonic corneoscleral graft and regular size penetrating keratoplasty or combined DALK and DMEK. In particular, the decentral trephination results in a bigger scleral area on one side of the donor transplant (Fig. 1C). In our case, we gained a 5.2-mm-diameter corneoscleral tectonic graft in this area (Fig. 1D, E) and used it successfully for covering a defect after block excision of a ciliary body tumor (Fig. 1F). With this novel approach, a peripheral graft of up to 7.5 mm diameter can be created, while—in contrast to (conventional) central donor trephination—the rest of the decentrally trephined donor transplant is still big enough to gain an up to 8.0-mm (Fig. 1D, E) central corneal graft. These central corneal grafts can be used for a regular-sized penetrating keratoplasty (here after perforated corneal herpetic ulcer; Fig. 1G) or for combined DMEK and DALK.

This is the most important advantage of this novel preparation technique. A single donor cornea might be used for up to three recipients, thus reducing the need for corneal tissue and—at least in the long run—limiting corneal donor tissue shortage [10]. Our case suggests that this approach seems to be feasible. Nonetheless, several questions have to be addressed. In some rare cases, the described split cornea technique might not be feasible, for example, if the preparation of the corneoscleral graft for the block excision results—contrary to preoperative expectations—in a too small corneal rest for the subsequent PK or combined DMEK and DALK. This might occur when a tectonic graft with a much larger diameter (i.e., > 7.5 mm) is necessary for covering defects after block excision of, e.g., ciliary body tumors, appearing larger intraoperatively than expected. In addition, the use of one single donor transplant for up to three patients assumes a specialized ophthalmology center performing tumor and corneal surgery as well as having sophisticated logistics in the surgical theater. Usually, surgeries using material from one single donor button are planned on the same day. After corneoscleral tectonic grafting, the remaining graft is stored in a closed organ culture system containing 100 ml minimal essential medium with 2% fetal calf serum, dextran, and antibiotics at 32 °C until preparation for keratoplasty. If it is not possible to perform the surgeries on the same day, the remaining donor material can be stored again in a closed organ culture system (maximum until the date of expiration). Therefore, also the eye bank needs sophisticated logistics including a functional documentation and labeling system.

Furthermore, these grafts have to be supplied by an external eye bank if the ophthalmology center is not having its own eye bank using this novel preparation technique.

For the sake of completeness, this novel preparation technique is not necessary for all kinds of corneoscleral grafting. For very small corneoscleral defects, corneoscleral buttons arising after standard trephination for PK, DMEK, or DALK could be used instead of being discarded.

However, in conclusion, this novel preparation technique of eccentric donor button preparation offers the opportunity to reduce the consumption of corneal donor tissue. Therefore, this modified trephination technique should be the standard approach in corneal donor transplant preparation if a block excision with tectonic corneoscleral graft is planned.
